# Computational Analysis of Constraints on Noncoding Regions, Coding Regions and Gene Expression in Relation to *Plasmodium* Phenotypic Diversity

**DOI:** 10.1371/journal.pone.0003122

**Published:** 2008-09-01

**Authors:** Kobby Essien, Sridhar Hannenhalli, Christian J. Stoeckert

**Affiliations:** 1 Department of Bioengineering, University of Pennsylvania, Philadelphia, Pennsylvania, United States of America; 2 Center for Bioinformatics, University of Pennsylvania, Philadelphia, Pennsylvania, United States of America; 3 Department of Genetics, School of Medicine, University of Pennsylvania, Philadelphia, Pennsylvania, United States of America; University of the Western Cape, South Africa

## Abstract

**Background:**

Malaria-causing *Plasmodium* species exhibit marked differences including host choice and preference for invading particular cell types. The genetic bases of phenotypic differences between parasites can be understood, in part, by investigating constraints on gene expression and genic sequences, both coding and regulatory.

**Methodology/Principal Findings:**

We investigated the evolutionary constraints on sequence and expression of parasitic genes by applying comparative genomics approaches to 6 *Plasmodium* genomes and 2 genome-wide expression studies. We found that the coding regions of *Plasmodium* transcription factor and sexual development genes are relatively less constrained, as are those of genes encoding CCCH zinc fingers and invasion proteins, which all play important roles in these parasites. Transcription factors and genes with stage-restricted expression have conserved upstream regions and so do several gene classes critical to the parasite's lifestyle, namely, ion transport, invasion, chromatin assembly and CCCH zinc fingers. Additionally, a cross-species comparison of expression patterns revealed that *Plasmodium*-specific genes exhibit significant expression divergence.

**Conclusions/Significance:**

Overall, constraints on *Plasmodium's* protein coding regions confirm observations from other eukaryotes in that transcription factors are under relatively lower constraint. Proteins relevant to the parasite's unique lifestyle also have lower constraint on their coding regions. Greater conservation between *Plasmodium* species in terms of promoter motifs suggests tight regulatory control of lifestyle genes. However, an interspecies divergence in expression patterns of these genes suggests that either expression is controlled via genomic or epigenomic features not encoded in the proximal promoter sequence, or alternatively, the combinatorial interactions between motifs confer species-specific expression patterns.

## Introduction

Every year, there are 350–500 million malaria infections and about 1 million people, primarily young children, die from malaria [1]. Members of the malaria-causing *Plasmodium* genus parasitize an extensive range of hosts including birds, rodents, reptiles and monkeys. These parasites share much similarity in their biology: (i) they are obligate intracellular parasites, (ii) they all have both a sexual and an asexual life cycle, and (iii) they all require an insect vector for transmission to their respective hosts. Yet there are striking differences between them. Including (i) different host preferences (e.g. *P. falciparum* infects humans whiles *P. berghei* is a rodent parasite), (ii) different insect vector preferences (*P. falciparum* is transmitted by *Anopheles gambiae* species while *P. berghei* is transmitted by *Anopheles dureni*), (iii) profound differences in the lengths of their various life stages (e.g. *P. falciparum* has a 48-hour life cycle in human erythrocytes while *P. berghei's* analogous cycle is 24 hours), (iv) morphological differences (*P. falciparum* gametocytes are banana-shaped while *P. berghei* ones are round or oval), and (v) cell preferences (*P. berghei* primarily invades immature red blood cells while *P. falciparum* does not discriminate between mature and immature ones). Additionally, some of the species such as the human parasites *P. vivax and P. ovale* possess an additional life stage in which they remain dormant in host liver. The sequencing of 8 *Plasmodium* genomes, 6 of which have been annotated, provides an opportunity to begin elucidating the molecular bases of the phenotypic differences between these parasites.

The genomic bases of phenotypic differences between species can be partly understood by investigating the constraints on coding regions, upstream regulatory regions and gene expression.

Coding regions may change as diverged species deal with disparate evolutionary pressures. Coding sequences of genes involved in processes such as sensory perception and gametogenesis have been shown to be under greater positive selection in human-chimpanzee comparisons [2]. The coding regions of transcription factors and other regulatory proteins have been found to evolve more quickly than those of proteins involved in core processes like metabolism and catalysis [3].

The fact that transcription factors diverge quickly between species supports the belief that the evolution of gene regulation is the major contributor to the phenotypic differences between species [4]. Genomic studies are beginning to shed light on the impact of regulatory changes on species diversity. A comparison of aerobic and anaerobic yeast species has revealed that the capacity for anaerobic growth is linked with the loss of the rapid growth element (RGE) in the promoters of mitochondrial ribosomal proteins [5]. Similarly, computational studies suggest that the difference in expression between *S. cerevisiae and C. albicans* methionine biosynthesis genes is due in part to the absence of GCN4 binding sites upstream of *C. albicans* orthologs [6]. Housekeeping genes have been observed to have less conserved upstream, putative regulatory, regions than other types of genes [7]. On the other hand, transcription factors, developmental genes and genes involved in processes such as cell communication and signalling tend to have more conserved upstream regions [8], [9].

The link between gene expression evolution and species diversity is reflected by results suggesting that the profiles of transcription factors change quickly between humans and non-human primates [10]. The importance of gene expression changes in diversity is further highlighted by the observation of a lack of conservation in expression relationships among human and chimpanzee cortical genes [11].

All these studies have been performed in yeast, *Drosophila* and higher eukaryotes. It remains to be seen whether their observations apply to *Plasmodium falciparum* with a unique lifestyle as an obligate intracellular parasite alternating between a sexual stage in an insect vector and asexual stages in a non-insect host. We performed an integrated study of constraints on protein coding regions, upstream regions and gene expression in *Plasmodium* species. We compared our results to those obtained from similar studies in other species and highlight ways in which *Plasmodium* differs from other species. Our analyses suggest that while some observations from other organisms, such as the tendency for transcription factors to be among the most divergent proteins, hold in *Plasmodium*, not all of them hold. Studies of proteins involved in the parasite's lifestyle may be more useful in understanding the phenotypic differences within the *Plasmodium* genus than studies of proteins involved in core eukaryotic processes.

## Results

### Analysis of protein coding regions

Systems-level analysis in mammals and other eukaryotes has led to the view that proteins involved in core processes like transport and metabolism as well as structural proteins have highly conserved coding regions. In contrast, regulatory proteins like transcription factors and kinases and proteins involved in developmental functions like gametogenesis are more diverged [2], [3]. To examine whether such a dichotomy in evolutionary rates existed in *Plasmodium* species, the ratios of nonsynonymous substitutions per nonsynonymous site to synonymous substitutions per synonymous site (dN/dS) for selected groups of proteins were examined in pairwise comparisons between *Plasmodium falciparum* and 5 other *Plasmodium* species (Table 1).

**Table 1:** *Plasmodium* genomes utilized in this paper, their status and sources.

Abbr.	Species	Version	Genome status	Source	Primary publication
*Pf*	*Plasmodium falciparum*	06/28/2007	Complete	PlasmoDB	[34]
*Pv*	*Plasmodium vivax*	09/11/2007	Complete	GenBank	Carlton et al., submitted.
*Pk*	*Plasmodium knowlesi*	02/22/2007	8×	GeneDB	Berriman et al., submitted.
*Py*	*Plasmodium yoelii yoelii*	09/10/2002	5×	PlasmoDB	[35]
*Pb*	*Plasmodium berghei*	02/27/2006	4×	PlasmoDB	[14]
*Pc*	*Plasmodium chabaudii*	02/27/2006	4×	PlasmoDB	[14]

The tendency of the proteins in a group of interest (e.g., transcription factors) to rank among the least constrained proteins (high dN/dS) among all proteins with orthologs in 2 species for any pairwise comparison was assessed using the Wilcoxon rank-sum test. The goal here was not to establish that particular groups of proteins had adaptively evolved but rather to rank groups by the level of constraint consequently we did not correct the *p-values* for multiple-testing as these were used for comparing groups rather than for establishing significance.

The 15 protein groups (Table 2) considered included metabolic processes (protein metabolism, macromolecule biosynthesis, mRNA processing, RNA splicing), transport (protein transport, ion transporter activity), structural proteins (structural molecule activity, structural constituent of ribosome, chromatin assembly), sexual development, transcription factors and kinases. Post-transcriptional regulatory mechanisms are thought to feature prominently in *Plasmodium* species so we also considered CCCH zinc fingers that are known to regulate mRNA decay and translation [12] and a set of *Plasmodium* orthologs of yeast and human mRNA decay proteins [13]. A set of cell invasion proteins was also considered because it is known that different *Plasmodium* species differ in their cell preferences.

**Table 2:** Protein groups analyzed, their sizes and sources.

Groups	Number of proteins	Source
Transcription factors	43	[26]; Proteins containing known DNA-binding domains (see Methods).
CCCH zinc fingers	16	Proteins containing CCCH domains (See Methods).
mRNA decay	30	[13]
Kinases	65	[36]
Cell invasion	87	[37]
Sexual development	246	[37]
Protein metabolism	849	[37]
Macromolecule biosynthesis	590	[37]
mRNA processing	149	[37]
RNA splicing	211	[37]
Protein transport	282	[37]
Ion transporter activity	491	[37]
Structural constituent of ribosome	124	[37]
Structural molecule activity	108	[38]
Chromatin assembly	92	[38]

**Figure 1:**
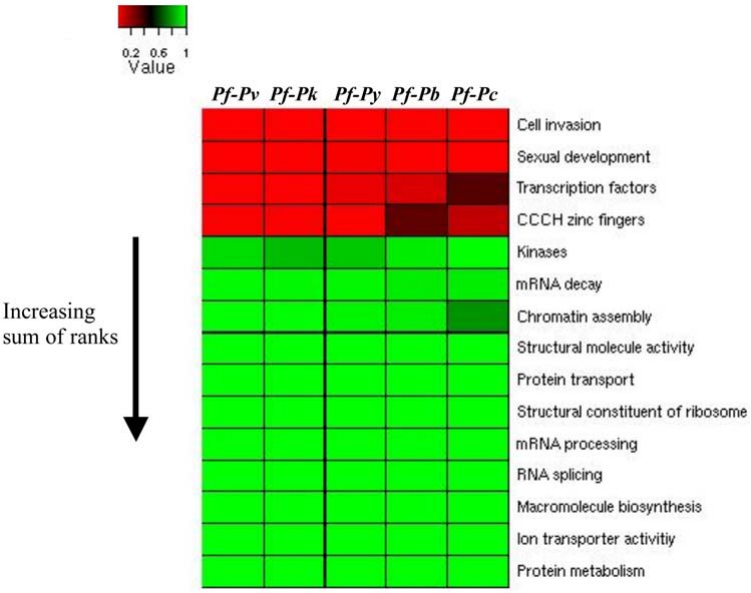
Gene groups arranged by level of constraint on coding regions. Cell invasion, sexual development, transcription factors and CCCH zinc fingers have less constrained protein coding regions than the other groups of genes considered. Elements of the heatmap represent the *p-values* resulting from testing the alternative hypothesis that proteins in a particular group rank among the least constrained proteins (higher dN/dS) among all proteins with orthologs in 2 particular species. The 15 groups were ranked from the least constrained to the most constrained in each of the 5 cross-species comparisons and the sum of the ranks was determined (Table S2) and used to order the protein groups in the heatmap. *Pf-Pv, Pf-Pk, Pf-Py, Pf-Pb* and *Pf-Pc* represent results from comparisons between *P. falciparum* and *P. vivax, P. knowlesi, P. yoelii, P. berghei* and *P. chabaudii* respectively.

For each of the cross-species comparisons, we ranked the groups of proteins in ascending order by the *p-value* resulting from the Wilcoxon rank-sum test and summed the ranks across species. A heatmap was then constructed with a row for each protein group (in increasing order of sum of ranks), a column for each cross-species comparison and color-coded entries representing the *p-values* from the Wilcoxon rank-sum test (Figure 1). Table S1 contains group sizes, median dN/dS' and *p-values*. The ranks and sum of ranks for the 15 groups are presented in Table S2. In all 5 cross-species comparisons, there is a clear partition between the low *p-values* of cell invasion, sexual development, transcription factors and CCCH zinc fingers on one hand and the other groups of proteins (Figure 1) and these 4 groups are consistently among the 5 least constrained groups (Table S2).

Proteins involved in core processes such as mRNA processing and protein metabolism exhibited extreme conservation suggesting that phenotypic differentiation between *Plasmodium* species, like differentiation between other eukaryotes, does not involve changes in the coding regions of these genes.

As observed in other eukaryotes, transcription factors were among the least constrained proteins across all species. In line with the notion that regulatory proteins are more diverged between species, the CCCH zinc fingers, which are important post-transcriptional regulators in *Plasmodium* species, also had low constraint. However, the kinases and components of the mRNA decay machinery, which are also involved in gene regulation, were quite constrained.

Both sexual development and cell invasion play direct roles in *Plasmodium's* parasitic lifestyle and are both ranked among the least constrained proteins. The low constraint between *Plasmodium* sexual development genes mirrors observations between human-chimpanzee gametogenesis genes [2]. Low constraint on cell invasion proteins reflects the key role signal peptide and transmembrane-containing proteins, both of which experience pressure from the host immune system, play in this process [14].

Overall, the behavior of *Plasmodium's* protein coding regions confirms observations from other eukaryotes in that transcription factors are under less constraint than proteins involved in core processes. However, unique aspects of *Plasmodium's* lifestyle influence the constraint-gene group relationship as genes involved in the specialized cell invasion process are also under less constraint.

To ensure that the results above were not artifacts of the evolutionary distance between *Plasmodium falciparum* and the 5 *Plasmodium* species considered, the analyses were repeated between all 10 pairwise combinations of *P. vivax*, *P. knowlesi, P. yoelii, P. berghei* and *P. chabaudii*. In all comparisons, transcription factors, zinc fingers, cell invasion and sexual development were among the 5 most divergent groups of proteins (Table S3).

### Analysis of upstream regions

We next sought to assess evolutionary constraints on promoter sequence by examining the tendency of orthologous promoters to share transcription factor binding motifs. We chose this approach as opposed to measuring promoter sequence divergence overall because (i) a motif-based approach does not rely on alignment accuracy while accounting for binding site turnover and (ii) it focuses on portions of the promoter that are more likely to be relevant to transcriptional regulation. As few transcription factor binding motifs have been identified in *Plasmodium* species we first generated a high confidence list of putative motifs. The list consisted of 63 motifs obtained by identifying 7-mers that were preferentially conserved in *P. falciparum* and *P. vivax* upstream regions and clustering them into position weight matrices using a previously published approach [15] (See materials and methods for details). Our list of putative transcription factor binding motifs included 5 of approximately 11 experimentally verified *P. falciparum* binding sites (Table 3) corresponding to a nominal sensitivity of 45%. These numbers are consistent with the fact that computational analyses of the *Plasmodium falciparum* genome has revealed approximately 43 transcription factors and currently only 50% of the proteome has been annotated. As the *Plasmodium falciparum* and *Plasmodium vivax* genomes have been completely sequenced while the other genomes are at 4×–8× coverage (Table 1), only the *P. falciparum* and *P. vivax* genome sequences were used for motif discovery.

**Table 3:** 5 motifs that are similar to known binding sites.

Motif consensus	Similar known site	Description of known site
A**CAGACA**	**CAGACA**GC	Binding site found upstream of sexual stage antigen, pgs28 [39].
**TGCACCC**	GG**TGCACCC**	Binding site of the AP2 transcription factor, PFF0200c [40].
**G**GG**TGCA**	**G**CA**TGCA**	Binding site of the AP2 transcription factor, PF14_0633 [40].
T**TGTAGT**	ACTGCA**TGTAGT**	Binding site found upstream of knob associated histidine-rich protein [41].
**AA**[A**G**]**GG**[A**G**]**A**	[**A**G]**NGGGG**[C**A**]	G-box found upstream of heatshock proteins [16].

Motifs are presented as consensus sequences. Square brackets signify that either of the enclosed nucleotides may occur at that particular position. An ‘N’ signifies that any nucleotide may occur at that particular position. Regions of similarity between known binding sites and predicted ones are in boldface.

The Jaccard index was used to assess the overlap in motif content between a pair of orthologous promoters. This metric (see materials and methods) normalizes the number of shared motifs by the union of the 2 numbers of motifs.

**Figure 2:**
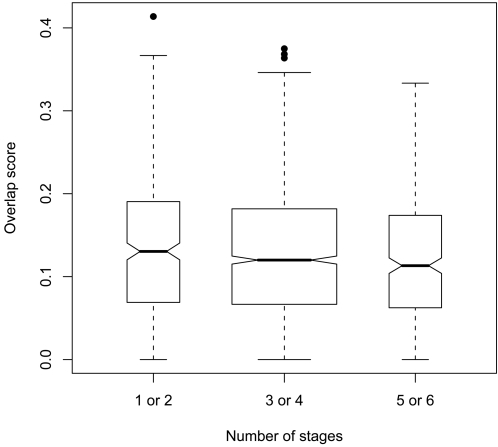
Motif overlap scores of genes present in particular numbers of *P. falciparum* expression stages. The distribution of *P. falciparum - P. vivax* motif overlap scores for genes present in 1 or 2, 3 or 4, and 5 or 6 stages suggests that genes present in fewer stages have more conserved upstream regions (more constrained upstream regions) than more broadly-expressed genes. Genes present in 1 or 2 stages have higher overlap scores than those present in 3 or 4 stages (Kolmogorov-Smirnov *p-value*: 0.036) and those present in 5 or 6 stages (Kolmogorov-Smirnov *p-value*: 0.013). The width of each box is proportional to the square root of the number of genes each box represents - 1 or 2 stages: 357 genes; 3 or 4 stages: 1411 genes; 5 or 6 stages: 354 genes. The upper edge of each box demarcates the overlap score at the 75^th^ percentile for genes present in a particular number of stages and the lower edge demarcates the overlap score at the 25^th^ percentile. The notch in each box occurs at the median overlap score. The lower whisker extends from overlap score at 25^th^ percentile to 0 and the upper whisker extends from the score at the 75^th^ percentile to the sum of the score at the 75^th^ percentile and 1.5 *(score at 75^th^ percentile–score at 25^th^ percentile). Dots above whiskers represent genes whose overlap scores lie beyond the upper whisker.

Previous studies have shown that in mammals, broadly-expressed house-keeping genes show less conservation in their promoters compared to tissue-restricted genes [7]. Farré and colleagues suggest that greater conservation upstream of tissue-specific and tissue-restricted genes may reflect a complex regulatory network. While single-celled organisms like *Plasmodium* lack tissues they have developmental stages and we tested the analogous hypothesis that genes present in more stages of the parasite's life cycle have less conserved upstream regions than those present in one or two stages in a *P. falciparum* - *P. vivax* comparison. As can be seen in Figure 2, genes present in more stages have smaller overlap scores than those present in fewer stages. Genes present in 1 or 2 stages had greater overlap scores than those present in 3 or 4 stages (Kolmogorov-Smirnov *p-value*: 0.036) and genes present in 1 or 2 stages had greater overlap scores than those present in 5 or 6 stages (Kolmogorov-Smirnov *p-value*: 0.013). Genes present in 3 or 4 stages have slightly but insignificantly larger motif overlap scores than those present in 5 or 6 stages (Kolmogorov-Smirnov *p-value*: 0.174). However, the overall trend suggests that upstream regions of stage-restricted genes exhibit greater conservation than the upstream regions of genes with less stage restriction.

Transcription factors and genes involved in development and cell communication have been observed to have higher promoter conservation than ribosomal proteins and proteins involved in processes such as catalysis and biosynthesis [8], [9]. Following the approach used earlier to examine constraints on coding regions, the 15 groups in Table 2 were examined for increased constraint on their upstream regions (greater conservation as determined by higher motif overlap) in pairwise comparisons between *P. falciparum* and each of the other 5 *Plasmodium* species.

**Figure 3:**
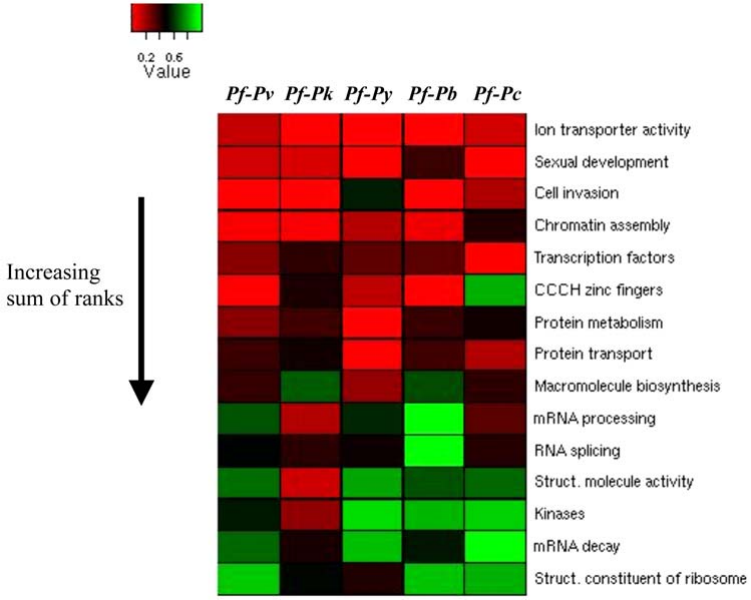
Gene groups arranged by level of constraint on upstream noncoding regions. Ion transport, sexual development, cell invasion, chromatin assembly, transcription factor and CCCH zinc finger genes have more constrained upstream regions than the other groups considered. Elements of the heatmap represent the *p-values* resulting from testing the alternative hypothesis that genes in a particular group rank among genes with the most constrained upstream regions (higher overlap scores) among all genes with orthologs in 2 particular species. The 15 groups were ranked from the most constrained to the least constrained in each of the 5 cross-species comparisons and the sum of the ranks (Table S6) was determined and used to order the gene groups in the heatmap. *Pf-Pv, Pf-Pk, Pf-Py, Pf-Pb and Pf-Pc* represent results from comparisons between *P. falciparum* and *P. vivax, P. knowlesi, P. yoelii, P. berghei* and *P. chabaudii* respectively.

Generally, upstream regions of ion transport, sexual development, cell invasion, chromatin assembly, transcription factor and CCCH zinc finger genes are under the greatest constraint (Figure 3). Genes involved in ion transport rank among the 5 most constrained groups of genes in all cross-species comparisons, cell invasion and sexual development genes rank among the 5 most constrained groups in 4 comparisons, chromatin assembly genes rank among the 5 most constrained in 3 comparisons and the transcription factors in 2 (Tables S5 and S6). As observed in other eukaryotes, upstream regions of transcription factors do exhibit increased constraint. However, there appears to be more striking constraint on the upstream regions of genes related to the parasite's unique lifestyle. *Plasmodia* are obligate intracellular parasites which upon invasion of red blood cells are surrounded by both a parasitophorous vacuole membrane (PVM) and the host cell membrane and consequently ion transporters are essential for processes such as nutrient uptake, metabolic waste removal and ionic homeostasis [16]. Sexual development is a critical function of these parasites as it is linked to the environmental shift from the host to insect vector. Cell invasion is a definitive feature of the parasite's lifestyle. *Plasmodium's* success as a parasite is partly due to epigenetic mechanisms that these parasites use to evade the host immune system. The chromatin assembly genes include Sir2, which is a known regulator of *Plasmodium's* host evasion system [17]. While not specifically related to *Plasmodium*'s parasitic functionality CCCH zinc fingers are important as they are over-represented in these species compared to other eukaryotes [12] suggesting that post-transcriptional regulation by these genes plays an important role in these parasites.

### Analysis of Expression

**Figure 4:**
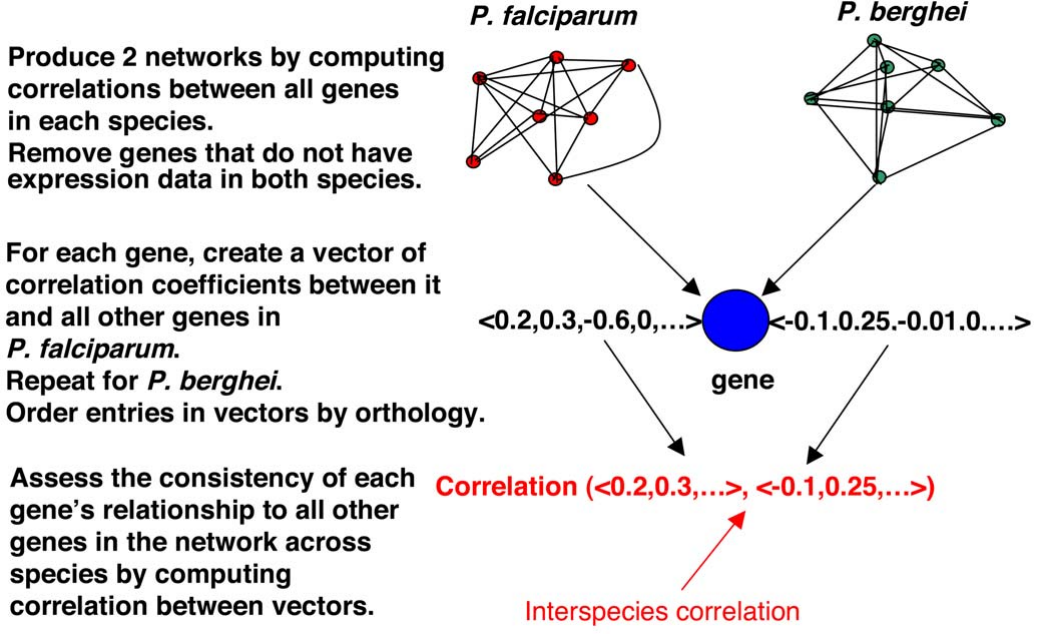
Estimating expression similarity across species. ‘Network-level’ interspecies correlation coefficients are estimated for genes with orthologs in both the *P. falciparum* and *P. berghei* expression datasets (see materials and methods for details).

A natural measure of expression-similarity between a pair of orthologous genes is the correlation between two expression profiles consisting of a set of ‘analogous’ samples. The *P. falciparum* expression dataset consists of 53 times points spanning the ring, trophozoite and schizont stages while the *P. berghei* dataset has only 4 points spanning the same stages. This difference in dimensionality makes it difficult to directly compare the expression of an orthologous pair of genes. Instead, we implemented a ‘network-level’ approach to estimate expression conservation (Figure 4). Briefly, to estimate the expression similarity between orthologs A and B, we compute the expression-similarity-vector E(A) representing similarity of A's expression with all other genes in the same species (likewise E(B)) and compute the correlation between E(A) and E(B) where the indices of the two vectors have 1-to-1 correspondence based on orthology relationships. The interspecies correlation of a gene is then estimated by assessing the similarity between vectors E(A) and E(B) (See materials and methods for details). Our approach is a modification of a technique previously used to compare gene expression between human and chimpanzee [11].

**Figure 5:**
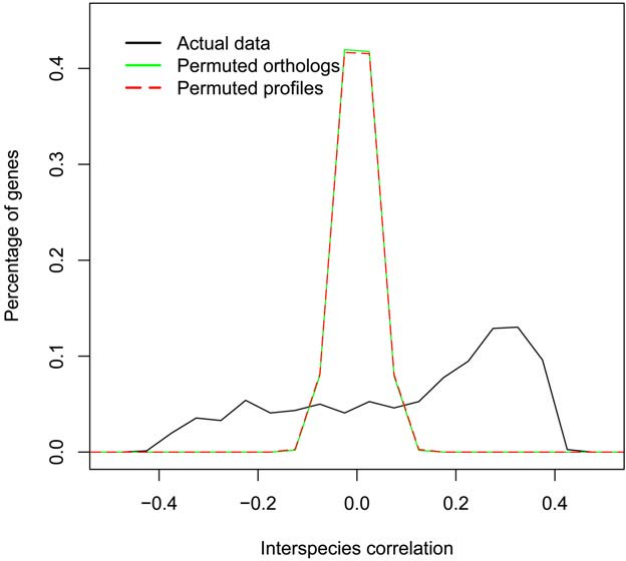
Interspecies correlations are non-random. A comparison of the actual distribution of interspecies correlations and 2 randomized distributions constructed from interspecies correlations computed from a 1000 permutations of the orthologous relationships and a 1000 permutations of expression profiles reveals that the interspecies correlations are non-random.

To establish that the interspecies correlations are non-random, the distribution of interspecies correlations was compared to 2 random distributions. The first random distribution was constructed by permuting the *P. falciparum* - *P.berghei* orthology relationships a 1000 times and computing the interspecies correlations for each of the permutations. The second was constructed analogously but with a 1000 permutations of both the *P. falciparum* and *P. berghei* expression profiles with the orthology mapping maintained. Both distributions of randomized correlations were approximately normally distributed around 0 while that of the actual data peaked at 0.4 but had a significant tail extending towards −0.4 (Figure 5). There was a statistically significant difference between each of the randomized distributions and the distribution of real interspecies correlations (Two-sided Kolmogorov-Smirnov *p-value* <2.2*10^−16^ for both the permuted ortholog and permuted profile distributions). The range of coefficients for the real data extended from −0.402 to 0.402 while the random data spanned the range −0.156 to 0.161 and −0.165 to 0.167 for the permuted orthologs and permuted profiles respectively. Also, 50% of the genes in the actual data were more conserved than the most conserved genes in the randomized datasets and 17% of the genes appeared more diverged than the most diverged genes in the randomized datasets.

**Figure 6:**
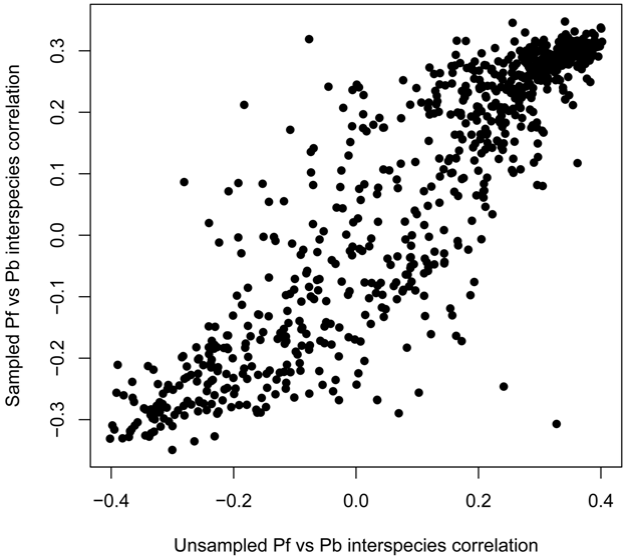
Interspecies correlations are not affected by differences in dimensionality between datasets. A comparison of the interspecies correlation coefficients obtained from the *P. berghei* and the unsampled and sampled *P. falciparum* expression datasets suggests the difference in dimensionality between the full *P. falciparum* and *P. berghei* datasets should not influence the results. The two sets of interspecies correlations have a Spearman rank-rho of 0.89 (*p-value* <2.2*10^−16^).

As our comparison involved computing interspecies correlations between a 53-time point *P. falciparum* and a 4-time point *P. berghei* dataset it was essential to establish that the difference in dimensionality would not significantly affect any inferred results. We computed the interspecies correlation coefficients using a sampled 4-time point *P. falciparum* dataset and the original *P. berghei* dataset and compared the resulting interspecies correlations to those obtained using the unsampled *P. falciparum* data. The sampled dataset was constructed by using maximum Spearman rank-correlation coefficients to map the best matching ring, trophozoite, young schizont and mature schizont time points between the original *P. falciparum* and *P. berghei* datasets. The *P. falciparum* - *P. berghei* interspecies correlation coefficients obtained using the unsampled and the sampled *P. falciparum* datasets were strikingly similar (Figure 6) with a Spearman rank-correlation of 0.89 (*p-value* <2.2*10^−16^).

**Figure 7:**
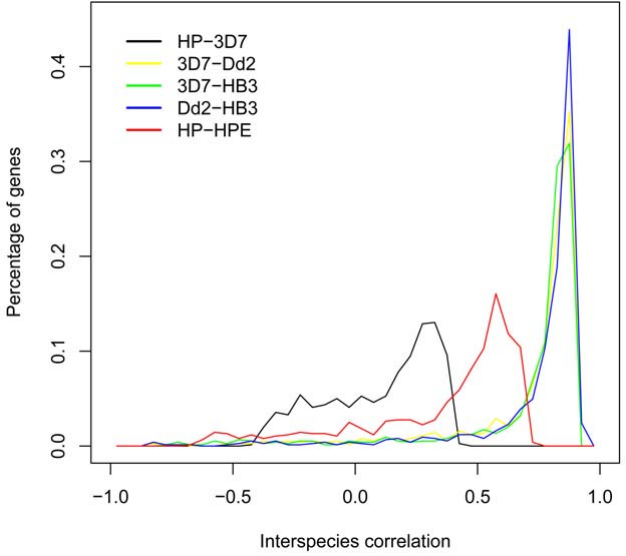
Interspecies correlations show a greater percentage of diverged genes than intraspecies correlations. There are significantly more diverged genes in the interspecies comparison (black: *P. falciparum* 3D7 strain vs *P. berghei* HP strain) than in the intraspecies comparisons (yellow: *P. falciparum* 3D7 strain vs *P. falciparum* Dd2 strain; green: *P. falciparum* 3D7 strain vs *P. falciparum* HB3 strain; blue: *P. falciparum* Dd2 strain vs *P. falciparum* HB3 strain; red: *P. berghei* HP strain vs *P. berghei* HPE strain).

Within a population of individuals from the same species there is a certain amount of naturally occurring expression divergence. To ensure that expression divergence signified by low interspecies correlations was outside the range of expected variation between members of the same species, we computed the intraspecies correlations of the 760 *P. falciparum* genes utilized in our initial analysis using microarray studies of the 3D7, Dd2 and HB3 strains of *P. falciparum* [18]. We also computed the intraspecies correlations of the 760 *P.* berghei genes used in the initial analysis utilizing *P. berghei* HP and HPE strains [14]. It can be seen from a comparison of these distributions that the interspecies correlations produce the largest percentage of genes with negative correlations (Figure 7). Specifically, 32% of genes in the HP-3D7 interspecies comparison had negative correlations whiles 5.0%, 5.6%, 4.0% and 16.2% of the genes in the 3D7-Dd2, 3D7-HB3, Dd2-HB3 and HP-HPE intraspecies comparisons respectively had negative correlations.

A particularly interesting protein that was discovered to be expression-diverged was Sir2 (interspecies correlation −0.231). *Plasmodium falciparum's* success as a parasite is partly due to its ability to antigenically vary the members of the 60-protein *P. falciparum* erythrocyte membrane protein 1 (PfEMP1) family. Only one PfEMP1 form is expressed at a time on the surface of an infected red blood cell and as the immune system mounts an attack on the expressed protein, the parasite switches to another member of the PfEMP1 family. Sir2 is a major component of the chromatin-silencing complex that regulates the variation of PfEMP1 [17]. *P. berghei* has families of antigenically variant proteins but lacks PfEMP1 orthologs so the divergence in expression of Sir2 likely reflects *P. berghei's* utilization of Sir2 to regulate a different protein family. It was noted earlier that the chromatin assembly group, to which Sir2 belongs, ranks among the groups of proteins with the most conserved upstream regions (Figure 3). The observation that a gene has greater upstream conservation in terms of promoter motifs but divergent interspecies expression raises the possibility that combinatorial interactions between motifs are partially conserved between species and small differences in the resulting cis-regulatory modules confer species-specific expression patterns or perhaps the gene's expression is not controlled via features in the proximal promoter.

As transcription factors bind to motifs in regulatory upstream regions to modulate gene expression it is reasonable to expect a relationship between motif overlap and interspecies correlation. However, we found that the two measures were not significantly correlated (Spearman rank-correlation 0.038 with a *p-value* of 0.156; n = 698). This is unsurprising given the complexity of gene regulation (involving epigenetic modifications and mRNA decay), as well as potential false positives in our binding site prediction. Also, a recent report suggests that the relationship between expression divergence and promoter divergence can be most clearly examined in controlled settings such as studies of gene expression responses in specific conditions known to be regulated by a few transcription factors [19].

**Figure 8:**
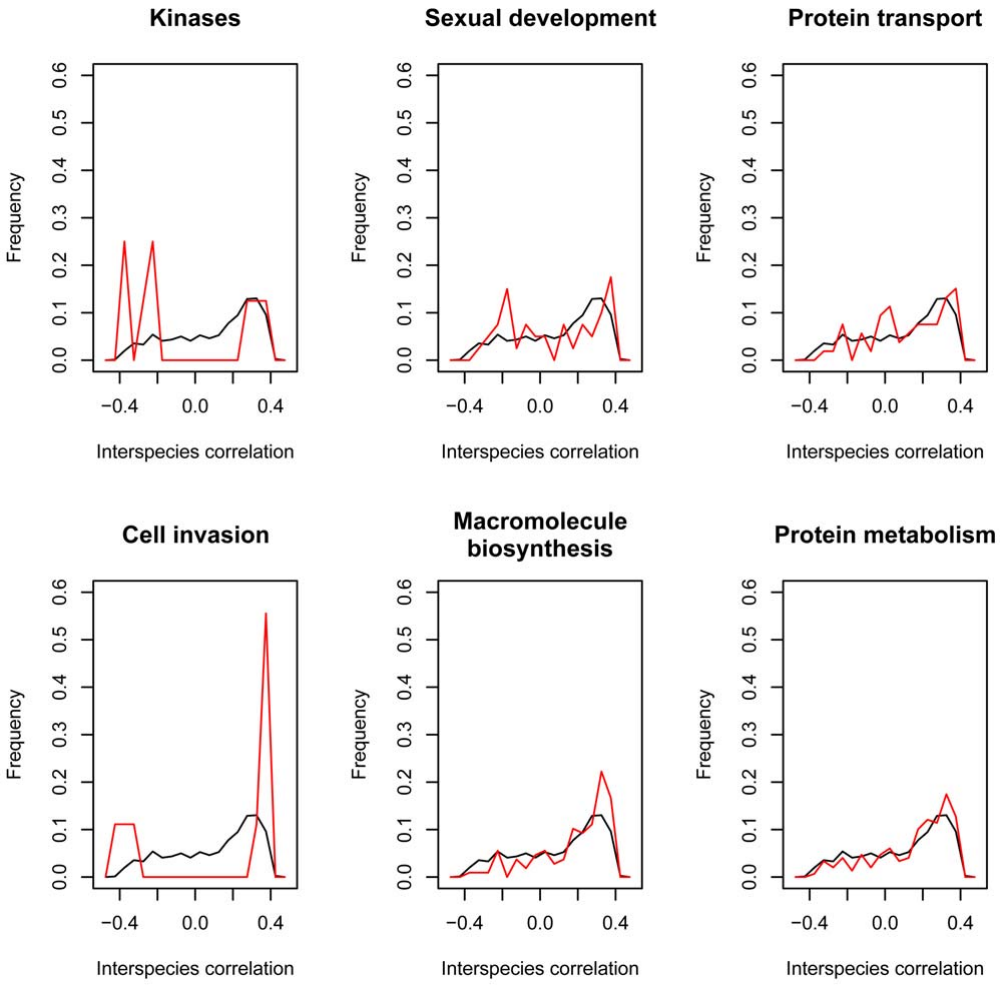
Distributions of interspecies correlation coefficients for 6 groups of genes. The distribution for each group is in red and that of all genes for which we have expression is shown in black (n = 760). Kinases (n = 9), sexual development (n = 41), protein transport (n = 54), cell invasion (n = 10), macromolecule biosynthesis (n = 109), protein metabolism (n = 150).

In primates, transcription factor genes have been observed to diverge in expression while in *Drosophila* species transcription factor gene expression evolves more slowly than those of other genes [10]. We assessed the tendency of our 15 gene groups of interest to rank among the most diverged genes using the Wilcoxon-rank sum test. We were unable to establish whether *Plasmodium* transcription factor genes were more expression-diverged than expected possibly due to the low number of transcription factor genes with orthologs in both expression datasets (Table 4). The *p-value* of the kinases is below 0.05 and it is the only group with a negative median interspecies correlation. Examination of the distribution of the interspecies correlations of groups with various *p-values* (Figure 8) showed that the kinases have a trimodal distribution of interspecies correlations with 6 of 9 proteins being diverged. Interestingly 4 of the 6 expression-diverged proteins have orthologs only in other apicomplexa, 1 has orthologs in apicomplexa, tetrahymena and plants and algae and 1 has orthologs in apicomplexa and entamoeba. None of the expression-conserved proteins have a phyletic distribution suggesting restriction to apicomplexa or closely related groups (Table 5). This raises the possibility that different parasites may differentially utilize phylum-specific kinases for regulatory purposes.

**Table 4:** Tendency of genes to rank among the most expression-diverged genes with data in both species.

Group	*Pf/Pb*		
	n	median	*p-value*
**Regulation**			
Transcription factors	7	0.088	0.351
CCCH zinc fingers	2	0.149	0.654
mRNA decay	3	0.256	0.699
Kinases	9	−0.226	**0.045**
**Lifestyle**			
Cell invasion	10	0.352	0.957
Sexual development	41	0.111	0.283
**Metabolism**			
Protein metabolism	150	0.213	0.998
Macromolecule biosynthesis	109	0.245	1.000
mRNA processing	29	0.297	0.999
RNA splicing	44	0.292	1.000
**Transport**			
Protein transport	54	0.161	0.799
Ion transporter activity	80	0.175	0.726
**Structure**			
Struct. constituent of ribosome	24	0.305	1.000
Struct. molecule activity	21	0.299	1.000
Chromatin assembly	10	0.340	0.974
**All genes**	760	0.169	

The alternative hypothesis that genes in a group rank among the most diverged (i.e. lower interspecies correlation) was evaluated using the Wilcoxon rank-sum test. The *p-value* of the most expression-diverged group is shown in boldface.

**Table 5:** Phyletic distribution of the 9 kinases for which interspecies correlations exist.

Protein	Api-specific	Plants, algae	Yeast	Metazoans	Other
**MAL7P1.73**	X				
**PF14_0320**	X				
**PF14_0227**	X				
**MAL13P1.84**	X				
**PF13_0085**					1
**PF11_0242**		X			2
PF11_0227		X	X		3,4
PF11_0096		X	X	X	1,2,3,4,5
PF13_0258		X		X	2,4

Proteins with diverged expression are in bold face. Kinases with diverged expression tend to be restricted to apicomplexa. Other column: 1- entamoeba, 2- tetrahymena 3- giardia, 4- dictyostelium, 5-kinetoplastids.

**Figure 9:**
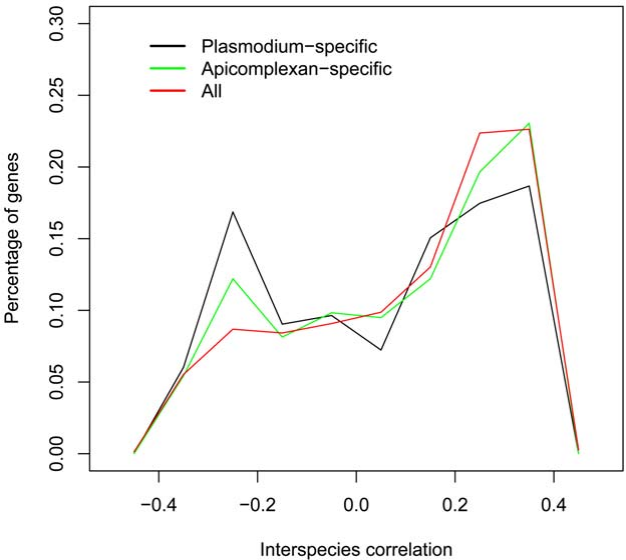
*Plasmodium*-specific genes are more likely to be diverged in expression. Comparison of interspecies correlations of all genes to that of apicomplexan- and *Plasmodium*-specific genes suggests that phyletically-restricted sets of genes are more likely to be diverged in expression. The distribution of all genes is shown in red (n = 760), that of apicomplexan-specific genes in green (n = 295) and that of *Plasmodium*-specific genes in black (n = 166).

To explicitly explore the relationship between expression divergence and the phyletic distribution of genes, apicomplexan- and *Plasmodium*-specific genes were obtained using PlasmoDB's phyletic profile query (http://plasmodb.org/plasmo/showQuestion.doquestionFullNameGeneQuestions.GenesByOrthologPattern) and the distributions of interspecies correlations for the corresponding genes examined. 295 of 2567 apicomplexan- and 166 of 1744 *Plasmodium*-specific genes had interspecies correlation data and comparison of the distributions of interspecies correlations of these groups of genes suggested a tendency for these restricted groups of genes to exhibit increased levels of expression divergence (Figure 9). Testing the alternative hypothesis that apicomplexan-specific genes were more expression-diverged than more phyletically-unrestricted genes resulted in a *p-value* of 0.115 using the Kolmogorov-Smironov test while the alternative hypothesis that *Plasmodium*-specific genes were more diverged than phyletically-unrestricted genes resulted in a *p-value* of 0.003. Removing genes whose expression profiles consist entirely of samples which are among the lowest 5^th^ percentile and 10^th^ percentile of genes assayed in a particular sample in either species and then re-testing the hypothesis that *Plasmodium*-specific genes were more expression-diverged than more phyletically-unrestricted genes resulted in *p-values* of 0.013 and 0.003 respectively. This suggests that observed the link between expression divergence and specificity to *Plasmodium* species is not merely the result of comparing genes with unresponsive spots on one microarray to genes with responsive spots on another.

## Discussion

As a preliminary step towards understanding genomic and transcriptomic determinants of phenotypic differences between *Plasmodium* species, we have performed a comprehensive analysis of constraints on protein coding regions, upstream regions and gene expression between *Plasmodium* species. We compared our results to those obtained from analogous studies in other organisms. Our results suggest that while some similarities exist between *Plasmodium* and other eukaryotes with regards to the groups of genes that are or are not constrained across related species, genes linked to the parasite's unique lifestyle as an obligate intracellular parasite capable of sexual development have constraints which suggest that these genes are important to diversity within the genus.

As observed in primates, the coding regions of *Plasmodium* transcription factors and developmental processes are less constrained than those of core processes such as metabolism and transport. However, *Plasmodium* cell invasion proteins and CCCH fingers are also under less constraint. Many cell invasion proteins are involved in host-pathogen interactions and consequently the reduction in constraint on the protein coding sequence may be a result of pressure from the host's immune system [14]. If it is generally true that regulatory proteins are among the most divergent proteins in species, it is reasonable to expect that the CCCH zinc fingers have less constraint on their coding regions as post-transcriptional regulation is thought to feature prominently in *Plasmodium* species [12].

Genes present in fewer stages of the parasite's life cycle have more constrained upstream regions than broadly-expressed genes. The promoters of mammalian tissue-restricted genes are more conserved than those of broadly-expressed genes [7] suggesting that even though *Plasmodia* are unicellular, similar principles govern the level of constraint on regulatory upstream regions of *Plasmodium* genes with varied gene expression distributions.

*Plasmodia* are similar to mammals in that transcription factors and developmental genes have more constrained upstream regulatory regions [8], [9]. However *Plasmodium* species extend this constraint to ion transport, cell invasion, chromatin assembly and CCCH zinc finger proteins, all of which are linked to the parasite's unique lifestyle.

Possibly due to limited expression data, we were unable to determine whether *Plasmodia* are similar to primates where the expression of transcription factors has quickly evolved or *Drosophila* species in which transcription factor expression appears conserved [10]. However, kinases that are specific to apicomplexa are more likely to be diverged in expression between *Plasmodium falciparum* and the rodent parasite *Plasmodium berghei* than those that occur in other phyla. It was also observed that in general *Plasmodium*-specific genes were more expression-diverged than phyletically-unrestricted ones. Many *Plasmodium*-specific genes reflect the genus' parasitic lifestyle and so it is possible that disparate members of *Plasmodium* genus modify the expression of parasite-specific genes to ensure that different species are successful in their various hosts and vectors.

In mammals, positively selected genes have highly conserved upstream regions suggesting that they are under tight regulatory control [8]. While our work did not entail identifying positively selected *Plasmodium* genes, the genes with the least constraint on their coding regions included the regulatory transcription factors and CCCH zinc fingers and parasite lifestyle-associated cell invasion and sexual development genes. These gene groups had constrained upstream regions suggestive of tight regulatory control. While ion transport and chromatin assembly genes, which are indirectly related to the lifestyle of these parasites, have constrained coding regions, they also have highly constrained upstream regions implying that their action in *Plasmodium* species requires tight regulatory control.

Generally, genes specific to *Plasmodium* species, many of which are important to the genus' unique lifestyle seemed more likely to be diverged in expression. The observations that lifestyle genes exhibit greater upstream conservation and more diverged expression are not contradictory as it is possible that lifestyle genes may be regulated by genomic and epigenomic mechanisms that only partially rely on sequence features within proximal promoters. Alternatively, the conservation of motifs upstream of lifestyle genes could be the result of partially conserved transcriptional modules that have differentially diverged across species. For example, one can imagine a case where an orthologous gene is regulated by 4 promoter motifs in both of 2 species with only 3 of those motifs conserved upstream of that particular gene. Despite the high upstream conservation conferred by 3 the motifs the difference of 1 motif may cause expression divergence. This would imply that the motif composition of transcriptional modules is quite important in *Plasmodium*. It has actually been suggested that the paucity of transcription factors and motifs in *Plasmodia* is a reflection of a greater reliance on combinatorial regulation via cis-regulatory modules in the genus than in other unicellular organisms such as yeast [20].

In this work we only examine constraints on coding sequence, upstream sequence and gene expression but to fully understand how *Plasmodium* species differ from each other, data from mRNA decay, epigenomic, proteomic and other types of genome-scale studies will be necessary.

Overall, constraints on *Plasmodium's* protein coding regions confirm observations from other eukaryotes in that transcription factors are under relatively lower constraint. Proteins relevant to the parasite's unique lifestyle also have lower constraint on their coding regions. Greater conservation between *Plasmodium* species in terms of promoter motifs suggests tight regulatory control of lifestyle genes. However an interspecies divergence in expression patterns of these genes suggests that either expression is controlled via genomic or epigenomic features not encoded in the proximal promoter sequence, or alternatively, the combinatorial interactions between motifs confer species-specific expression patterns.

## Materials and Methods

### Data

*P. falciparum* and *P. berghei* expression datasets [18], [21], [14] were obtained from PlasmoDB [22]. Genome sequences were downloaded from the appropriate databases (see Table 1). Orthologs were retrieved from OrthoMCL-DB Version 2 [23].

The stage distribution of genes was determined by counting the stages each gene was detected in during a study of the blood, sporozoite and gametocyte stages of the parasite [24]. Genes whose total sum of MOID scores across the 6 stages was less than 200 were removed from the dataset and a gene was noted as detected in a particular stage if its MOID score in that stage was 10% or more of the total sum of its MOID scores across the 6 stages [25].

### Supplemental identification of regulatory proteins

In addition to the AP2 transcription factors [26], *Plasmodium falciparum* is known to contain proteins with the ARID, AT-hook, C2H2 zinc finger, Myb, Tubby, TATA-binding and CAAT-box DNA-binding domains [12]. *Plasmodium falciparum* proteins containing these domains were identified by using HMMER to scan the *Plasmodium* proteome with the markov models of the above domains from the Pfam Protein families database [27]. A protein is taken to contain a domain if its E-value exceeds the domain-specific Pfam-defined gathering threshold. These transcription factors are presented in Table S7.

CCCH zinc finger-containing proteins were identified by scanning the *Plasmodium* proteome with CCCH markov model. The identified CCCH zinc finger proteins are given in Table S7.

### Estimation of protein evolutionary rates

Evolutionary rates of protein coding regions were computed with *codeml* [28], [29]. Codon equilibrium frequencies were estimated from the average nucleotide frequencies at 3-codon positions. The Nei-Gojobori method was used in determining the substitution rates. Prior to analysis, alignments were performed with *Clustalw* [30] and gaps and ambiguous characters were removed from alignments.

### Estimation of promoter conservation via assessment of sharing of binding sites

We generated a high confidence list of putative binding sites using an approach previously utilized in identifying motifs in mammals [15]. We first aligned orthologous *P. falciparum* and *P. vivax* genes and their upstream regions using *lagan* [31]. *P. falciparum* sequence was masked for low complexity regions using *dust* prior to analysis. It was not necessary to mask the *P. vivax* sequence as removing low complexity regions in *P. falciparum sequence* will prevent *P. falciparum* low complexity regions from aligning with orthologous *P. vivax* low complexity regions. The aligned region 2000 bp upstream of each gene (or until the nearest upstream gene if that was closer) was then extracted and used in subsequent analysis. Regions of 10 bp or more with at least 50% identity were classed as conserved and were searched for 7-mers that were conserved at a higher rate than expected based on conservation rates of 7-mers of the same GC-content. For each k-mer *x* the conservation rate is computed as
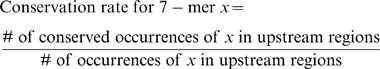
An expected number of conserved instances for each 7-mer *x* is then estimated by taking the product of the number of occurrences of 7-mer *x* and the average conservation rate of 20 7-kmers with the same base composition as *x*.
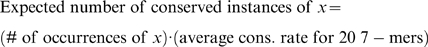
It is critical to take a 7-mer's base composition into account when computing its expected conservation rate as *Plasmodium falciparum's* genome is highly AT-rich and without this measure the analysis will likely be biased towards AT-rich 7-mers.

Finally, a conservation score (z-score) is computed as
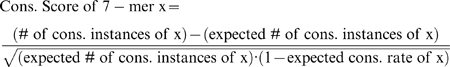
Statistics of discovered 7-mers are given in Table S4.

One of each 7-mer - reverse complement pair was then removed to avoid double-counting as both strands of upstream sequence were searched for 7-mers. Thresholding the resulting list at a conservation score of 2.5 resulted in a set of 130 7-mers. To account for redundancy within 7-mers, they were ranked by conservation score and as the 7-mer list was traversed in descending order 7-mers with 1 mismatch or an overlap of 6 consecutive bases to an already traversed 7-mer were clustered together [32]. The resulting clusters are presented in Text S1. The clustering gave rise to 63 motifs that were represented as position weight matrices (PWMs).

The region 2000 bp upstream of each gene (or until the nearest upstream gene if that was closer) was scanned in each species independently with each of the 63 PWMs. All upstream regions were masked for low complexity regions with *dust* prior to scanning. Scanning was performed with PWM_SCAN [33] at a *p-value* cutoff of e^−8.5^, which corresponds to a random expectation of 1 hit every 5000 bp.

We assessed the tendency for orthologous upstream regions to share motifs using the Jaccard index as an overlap score. The Jaccard index between an orthologous upstream region *X* from 2 species for example, *P. falciparum* and *P. vivax,* is computed as:
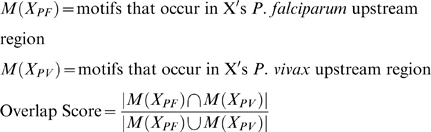
The score ranges from 0 in the case of no overlap to 1 in case of exact overlap.

### Expression analysis

We sought to identify genes whose transcriptional relationship to other genes in the coexpression network had changed between the 3D7 strain of *P. falciparum* and HP strain of *P. berghei*. A coexpression network was first created for each species by computing the Pearson correlation coefficients of all genes in each expression dataset. Genes that lacked orthologs in both datasets were then removed from each of the two networks. In each network a vector was constructed for each gene containing the correlation coefficients between that gene and all other genes in the network. Orthologous vectors are constructed such that corresponding positions contain coexpression information for the same pair of genes. The similarity of a gene's relationship to all other genes in its network is then compared across species by computing the Pearson correlation between the gene's orthologous vectors. We refer to this final similarity metric as a gene's interspecies correlation and it can range from −1, signifying that a gene's relationship to other genes in the network has drastically diverged to +1, showing that a gene's relationship to all other genes in the network is extremely conserved between the two species. This analysis is a modification of an approach used by Oldham and colleagues to examine coexpression networks in human and chimpanzee brains [11]. Computation of the interspecies correlation is outlined pictorially in Figure 4. A total of 760 genes had expression data in both datasets and their interspecies correlations are given in Table S8.

## Supporting Information

Table S1 Assessment of the tendency of proteins to rank among the least constrained proteins with orthologs in 2 species. The alternative hypothesis that the proteins in a group rank among the least constrained (high dN/dS) was tested using the Wilcoxon rank-sum test. The *p-values* of the 5 least constrained groups of proteins are shown in bold face. In all comparisons, transcription factors, zinc fingers, cell invasion and sexual development proteins are among the least constrained proteins. This table only includes data for the 5 comparisons used to construct Figure 1.
(0.04 MB XLS)

Table S2 Group rankings based on the level of constraint on protein coding regions as determined by the Wilcoxon rank-sum test for each of the 5 cross-species comparisons presented in Table S1. The smaller the rank, the less the observed constraint. Protein groups are sorted by sum of ranks.
(0.03 MB XLS)

Table S3 Assessment of the tendency of proteins to rank among the least constrained proteins with orthologs in non-*falciparum* comparisons. The alternative hypothesis that the proteins in a group rank among the least constrained (high dN/dS) was tested using the Wilcoxon rank-sum test. The *p-values* of the 5 least constrained groups of proteins are shown in bold face. In all comparisons, transcription factors, zinc fingers, cell invasion and sexual development proteins are among the least constrained proteins. This table only includes data for all pairwise comparisons not involving *P. falciparum*. For analogous *P. falciparum* data see Table S1.
(0.08 MB XLS)

Table S4 Statistics of 7-mers discovered in *P. falciparum* 2000 bp upstream regions. The table contains all 7-mers discovered in *P. falciparum* 2000 bp upstream regions, their number of occurrences, their number of conserved occurrences, their conservation rates and conservation scores (z-scores).
(3.24 MB XLS)

Table S5 Assessment of the tendency of each gene group to rank among the genes with the most constrained upstream regions (high motif overlap) among all genes with orthologs in 2 particular species. The alternative hypothesis that genes in a group rank among the most constrained was tested with the Wilcoxon rank-sum test. The *p-values* of the 5 most constrained groups of proteins are shown in bold face. Ion transport, sexual development, cell invasion, chromatin assembly, transcription factor and CCCH zinc finger genes have more constrained upstream regions than the other groups of proteins considered. This table includes data used to construct Figure 3.
(0.04 MB XLS)

Table S6 Group rankings based on level of constraint on upstream regions as determined by the Wilcoxon rank-sum test for each of the 5 cross-species comparisons presented in Table S5. The smaller the rank, the greater the observed constraint. Gene groups are sorted by sum of ranks.
(0.03 MB XLS)

Table S7 Supplemental regulatory proteins identified based on domains. Proteins were identified by scanning the *Plasmodium* proteome with known regulatory domains from the Protein Families Database, PFAM.
(0.04 MB XLS)

Table S8 Interspecies correlations for *P. falciparum* genes with orthologous expression data in both 3D7 and HP.
(0.13 MB XLS)

Text S1 7-mer clusters used to create the 63 motifs. Each line in the file contains each of the clusters used to create the position weight matrices utilized in the paper. Note that 43 of the 63 clusters are singletons.
(0.00 MB TXT)
